# Intraoperative Hemodynamic Instability and Higher ASA Classification Increase the Risk of Developing Non-Surgical Complications following Orthopedic Surgeries

**DOI:** 10.3390/jcm13061689

**Published:** 2024-03-15

**Authors:** Ting-Jui Hsu, Jen-Yu Chen, Yu-Ling Wu, Yu-Han Lo, Chien-Jen Hsu

**Affiliations:** Department of Orthopedic, Kaohsiung Veterans General Hospital, Kaohsiung 813414, Taiwan; tjhsu1925@vghks.gov.tw (T.-J.H.); jychen@vghks.gov.tw (J.-Y.C.);

**Keywords:** orthopedic surgery, ASA classification, hemodynamic instability

## Abstract

(1) **Background:** Either pre-operative physical status or unstable hemodynamic changes has been reported to play a potential role in causing vital organ dysfunction. Therefore, we intended to investigate the impact of the American Society of Anesthesiologist (ASA) classification and intraoperative hemodynamic instability on non-surgical complications following orthopedic surgery. (2) **Methods:** We collected data on 6478 patients, with a mean age of 57.3 ± 16, who underwent orthopedic surgeries between 2018 and 2020. The ASA classification and hemodynamic data were obtained from an anesthesia database. Non-surgical complications were defined as a dysfunction of the vital organs. (3) **Results:** ASA III/IV caused significantly higher odds ratios (OR) of 17.49 and 40.96, respectively, than ASA I for developing non-surgical complications (*p* < 0.001). Non-surgical complications were correlated with a 20% reduction in systolic blood pressure (SBP), which was intraoperatively compared to the pre-operative baseline ((OR) = 1.38, *p* = 0.02). The risk of postoperative complications increased with longer durations of SBP < 100 mmHg, peaking at over 20 min ((OR) = 1.33, *p* = 0.34). (4) **Conclusions:** Extended intraoperative hypotension and ASA III/IV caused a significantly higher risk of adverse events occurring within the major organs. The maintenance of hemodynamic stability prevents non-surgical complications after orthopedic surgeries.

## 1. Introduction

Aside from instances related to the surgical technique, perioperative complications for patients undergoing orthopedic surgery are not uncommon. In a nationwide database study involving 146,774 patients in the USA conducted by Cesar S. Molina et al., the overall complication rate for orthopedic surgery was reported to be 5% [1]. Consequently, a considerable number of studies concerning the risk factors for developing complications were published in an attempt to provide a policy for preventive measures and early management.

Medical co-morbidities are essentially precipitating factors for developing perioperative complications. Therefore, a higher rate of medical co-morbidities among geriatric patients was reasonably assumed to amplify the risk of perioperative complication associated with orthopedic surgeries, especially for patients over 65 years old [1].

The American Society of Anesthesiologists’ physical status classification system (ASA classification) is commonly utilized as a tool for evaluating the overall health statuses of patients. Most anesthesiologists recognize its capacity to forecast both short- and long-term complication rates in patients undergoing surgery [2,3,4,5]. From the viewpoint of pathophysiology, intraoperative hemodynamic instability could compromise the blood supply to vital organs, like the brain, heart, lungs, liver, and kidneys. Consequently, hemodynamic instability was inevitably attributed to the development of postoperative complications in various surgical procedures [6,7,8,9,10]. In contrast to ASA classification, which is an intrinsic factor that exists pre-operatively, hemodynamic instability seems to be an extrinsic factor that can be treated intraoperatively. Although anesthesiologists treat hemodynamic instability, the threshold and duration of hemodynamic fluctuations remain an issue, generating discussions about whether the postoperative complications related to hemodynamic instability cause the dysfunction of major organs or even mortality [6,9,11].

The primary objective of this retrospective single-center study was to explore the associations between the development of postoperative medical complications and either pre-operative ASA classification or intraoperative hemodynamic changes. Subsequently, we intended to clarify the impact of hemodynamic changes on causing postoperative complications by delineating what thresholds and durations of hemodynamic fluctuation induce major postoperative complications for patients undergoing orthopedic surgery.

## 2. Materials and Methods

With the approval of the Institutional Review Board, we explored a clinical database on anesthesia to collect data on study subjects who were operated on under general anesthesia (GA) and spinal anesthesia (SA) at the Department of Orthopedics from 2018 to 2020. The diagnoses were recorded using the ICD 10. The surgical procedures were confirmed and classified using coding defined by the Taiwan Health Insurance Administration. Therefore, we set the inclusion criteria as follows: patients older than 20 who were hospitalized for orthopedic operations performed under GA or SA.

We collected data on pre-operative demographic characteristics, including age, gender, ASA classification, biochemical data, and pre-operative blood pressure. Subsequently, we extracted their anesthetic course records, which were automatically recorded every 5 min using an anesthetic machine. These records included data on blood pressure, heart rate, and respiratory mode, along with respiratory rate settings. Adverse events were defined as complications based on extraordinary examination results or successive follow-ups of postoperative biochemical examinations. A complication involving the brain was defined as the detection of abnormal findings via computed tomography (CT) or magnetic resonance imaging. A complication involving the heart was determined by observing abnormal findings on an electrocardiogram (EKG) or a significant elevation in the cardiac enzyme levels. A complication related to the kidneys was characterized by progression to Stage 3 chronic kidney disease, with an estimated glomerular filtration rate of ≤59 mL/min 1.73 m^2^. A complication concerning the liver was defined as an elevation in the Glutamic Oxaloacetic Transaminase/Glutamic Pyruvic Transaminase value to at least four-times higher than the pre-operative baseline value. Lastly, a complication related to the lungs was identified via the need for ventilation for more than three days postoperatively or the presence of abnormal findings indicating pulmonary embolism via an EKG or CT scan.

We analyzed the major parameters by dividing the study course into pre-operative, intraoperative, and postoperative periods to clarify the impact of developing postoperative complications. We adopted descriptive statistics and logistic regression analysis data generated using SPSS version 20.3.

## 3. Results

**Figure 1:**
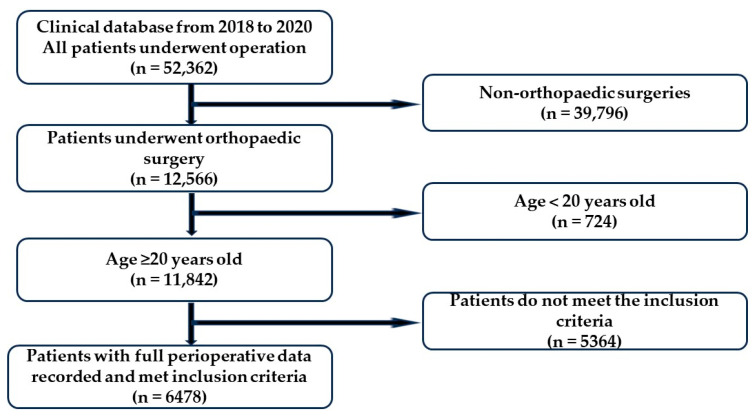
Flowchart of collecting the study subjects.

By exploring the Clinical Database of Anesthesia, we identified 12,566 patients who underwent orthopedic surgeries out of 52,362 patients operated on between 2018 and 2020. Considering the inclusion criteria, we collected data from 11,842 patients over 20 years old. After excluding patients with incomplete blood pressure records during operation, 8353 patients who underwent operations under GA or SA were included in the study. Finally, 6478 patients with a recorded ASA classification were enrolled (Figure 1). Among them, 388 patients (5.99%) developed significant postoperative complications (Table 1), with kidney- and heart-related complications comprising 53% (206 patients) and 42% (162 patients) of the total, respectively. The remaining complications included 14 cases of brain-related issues, 13 cases of lung-related issues, and 7 cases of liver-related issues.

The gender distribution of 6478 patients was 3571 females and 2907 males, with an average age of 57.28 ± 18.01 years old. However, the patients with postoperative complications exhibited a notably higher average age of 68.1 years (Table 1). In logistic regression analysis (Table 2), age as a parameter did not show an odds ratio (OR) warranting attention, despite having a significant statistical *p* value. In contrast, ASA classification (ASA III: (OR) = 17.49, *p* < 0.001; ASA IV: (OR) = 40.96, *p* < 0.001) and spinal anesthesia ((OR) = 1.68, *p* < 0.001) demonstrated a clear elevation in the odds ratio when comparing patients with and without postoperative complications (Table 2). We excluded other anesthetic methods except GA/SA in the original design of our study. We performed subgroup analysis (Table 3) of the patients administered GA/SA undergoing operations on the lower extremities, since surgeries on the spine and upper extremities were conducted under GA in our study. More non-surgical complications developed in the patients under spinal anesthesia than those under general anesthesia, even though the occurrence of hemodynamic instability episodes did not proportionally increase.

Since the anesthetic machine automatically recorded their blood pressure and heart rate every 5 min, we defined an event of hemodynamic instability as two successive records revealing systolic blood pressure (SBP) < 100 mmHg or mean arterial blood pressure (MAP) < 65 mmHg. This definition implies that the event persisted for at least 5 min, with SBP < 100 mm Hg or MAP < 65 mmHg. Regarding the impact of hemodynamic instability events, there was a trend that higher percentage of patients with postoperative complications occurred in cases of more hemodynamic instability events (Table 4). In Table 5, logistic regression analysis revealed that an odds ratio greater than 1.0 was observed for those with either more than two episodes of SBP < 100 mmHg or two or three episodes of MAP < 65 mmHg, while being compared to those without hemodynamic instability events. In spite of the above-mentioned findings, we noticed, paradoxically, that fewer complications occurred in patients with four or more episodes of MAP < 65 mmHg than in patients without MAP < 65 mmHg (Table 4). 

Considering the individualized, pre-operative baseline blood pressure, we investigated the changes in pre-operative and intraoperative blood pressure. We found a significantly elevated odds ratio ((OR) = 1.38, *p* = 0.02) when the reduction in blood pressure exceeded 20%, both pre-operatively and intraoperatively (Table 5).

Table 6 and Table 7 display the operation time and blood loss among patients without and with hemodynamic instability episodes with SBP < 100 mmHg/MAP < 65 mmHg according to whether they had operations on the upper extremities, lower extremities, or spine. For those who had operations on the lower extremities, more blood loss and a longer operation time caused more non-surgical complications.

Based on the aforementioned analysis, the preliminary results indicated significant parameters contributing to the development of postoperative complications, including older age, SA administration, a higher frequency of occurrences of SBP < 100 mmHg or MAP < 65 mmHg, and a reduction of SBP ≥ 20% between the pre-operative baseline and intraoperative measurements. Moreover, there was a noticeable increase in the odds ratio when comparing ASA IV and III to ASA I in terms of the likelihood of causing postoperative complications.

## 4. Discussion

In this study involving 6478 patients who underwent orthopedic surgery, 388 patients developed significant postoperative complications unrelated to the surgical process or technique. Apart from the ASA classification, which played a crucial role in contributing to a higher rate of complications (ASA III: (OR) = 17.49, *p* < 0.001; ASA IV: (OR) = 40.96, *p* < 0.001), neither gender nor age exhibited a notable difference among the pre-operatively recorded demographic characteristics. Concerning the parameters of the intraoperative course, the unfavorable factors included SA and a reduction of more than 20% in SBP during the intraoperative period compared to the baseline SBP ((OR) = 1.38, *p* = 0.02). We observed that more events of hemodynamic instability were associated with higher odds of developing postoperative complications.

Numerous studies have indicated robust associations between the ASA classification and postoperative medical complications, and even mortality, no matter what surgical procedures were conducted [2,3,5,9,12]. Hackett and colleagues analyzed data from over 2,000,000 patients who underwent various surgeries and discovered a progressive increase in the risk of complications ((OR) ranging from 2.05 to 63.25, *p* < 0.001) and mortality ((OR) ranging from 5.77 to 2011.92, *p* < 0.001) as the ASA classification ascended from II to V [5]. Vernooij et al. reported that 10,432 patients undergoing non-cardiac surgery with ASA III or IV comprised 58.1% of the postoperative complications; however, ASA III and IV comprised only 40.8% of the patients without a postoperative complication [9]. As a comprehensive assessment of their general condition, the ASA classification has consistently demonstrated the capacity to predict postoperative complications with inter-rater reliability while evaluating orthopedic trauma patients [13]. 

A previous study reported ASA classification as an effective predictive tool for postoperative complications among a wide spectrum of orthopedic surgical procedures. Several nationwide studies focusing on the association between the ASA classification, complications, and mortality yielded consistent outcomes. Meyer et al. observed that a higher ASA classification consistently correlated with elevated risks of causing complications [12]. Beecham et al. reported that 50% of 52 patients undergoing hip surgeries with ASA grade III or IV developed hemodynamic instability intraoperatively [14]. Our patients categorized as ASA III or IV also displayed an elevated odds ratio of developing postoperative complications of 17.49 or 40.96, respectively. ASA classification remains an important predictive tool for developing postoperative complications and the subsequent sequelae.

Our study demonstrated that more postoperative complications took place when more hypotensive events occurred, decreasing either the SBP or MAP. Currently, the duration and extent of perioperative episodes of hypotension causing postoperative complications are encouraging scientific investigations. Several previous studies have discussed the impact of hypotensive events during anesthesia administration on developing complications [6,7,8,9,11,14,15]; for instance, Vernooij et al. conducted a study involving 10,432 patients who underwent non-cardiac surgery. In this study, an increased number of episodes ((OR) = 1.02, *p* < 0.001) and a longer total duration ((OR) = 1.01, *p* < 0.001) of relative MAP decreasing by 20% from the baseline were significantly associated with an elevated risk of postoperative myocardial injury [9]. However, we disclosed, paradoxically, that fewer complications occurred in patients with four or more episodes of MAP < 65 mmHg than in patients without an MAP < 65 mmHg (Table 4). We also noticed that 4228 patients did not have an MAP < 65 mmHg, while only 66 patients sustained four or more episodes of MAP < 65 mmHg, respectively. We should not attribute this unexpected finding to the large difference in case numbers between the two groups until further analysis can support this deduction. However, more aggressive management by anesthesiologists is supposed to avoid the development of non-surgical complications when dealing with patients with more episodes of hemodynamic instability. Consequently, non-surgical complications did not necessarily occur if the episode of hemodynamic instability was handled properly in time.

In our other analysis, although statistical significance was not reached, we found that the odds ratio for postoperative complications increased with the following durations of hypotension: over 15 min of MAP < 65 mmHg ((OR) = 1.49, *p* = 0.27) and over 20 min of SBP < 100 mmHg ((OR) = 1.33, *p* = 0.34). In 2017, a Perioperative Quality Initiative consensus-building conference held in London concluded that even brief durations of SBP < 100 mmHg and MAP < 60–70 mmHg are harmful during non-cardiac surgery [16].

Hypotensive anesthesia is a common drug in orthopedic surgery for reducing intraoperative blood loss, minimizing transfusion requirements and optimizing surgical efficiency by enhancing the clarity of the surgical field. However, substantial variations in blood pressure are supposed to elevate the risk of postoperative complications. However, some authors have reported that a difference of SBP > 20% is not necessarily associated with the 30-day mortality rate or adverse outcomes [6,11]. This discrepancy may arise from differences in defining pre-operative standard blood pressure. In our study, we used the average blood pressure measured pre-operatively on a ward as the baseline to mitigate the influence of hypertension due to pre-operative anxiety and stress. A randomized controlled trial (RCT) implemented a personalized strategy for maintaining SBP within 10% of the baseline resting value [17]. This personalized strategy effectively realized lower rates of postoperative organ dysfunction. Therefore, individualized blood pressure management, particularly among surgical patients with poorly controlled hypertension, deserves recommendation. The prevention of substantial deviations from the baseline blood pressure level during anesthesia administration justifies tailored care for high-risk surgical patients. 

Regarding the thresholds for defining intraoperative hypotension, a considerable number of studies have proposed various suggestions [6,9,11]. Among these studies, no consensus was achieved regarding whether the threshold for MAP or SAP ranged from 50 to 100 mmHg or fluctuated from 10% to 40% in comparison with the baseline values. In a retrospective, registry-based cohort study between January 2015 and July 2016 involving 11,304 patients, the authors observed that an SBP below 80 mmHg and an MAP below 60 mmHg exhibited the strongest associations with the 30-day mortality rate, with odds ratios of 3.02 (95% confidence interval (CI) 1.81–5.07) and 3.77 (95% CI 2.25–6.31), respectively [11]. Furthermore, this study suggested that longer durations of hypotension were linked to more severe long-term complications. Our study also demonstrated that a longer hypotensive duration was an unfavorable factor for developing complications in the clinical context. We also discovered that a relative difference in the pre-operative and postoperative SAP over 20% caused a higher rate of postoperative complications. Interestingly, one study observed 42,825 anesthetized patients to find that intraoperative hypotension occurred in 30,423 (71%) patients, including 22,569 (53%) patients before skin incision and 24,102 (56%) patients after incision. Therefore, they recommended that anesthetists should avoid MAP < 65 mmHg during surgery [18].

Our analysis revealed a significantly higher postoperative complication rate in the SA group compared to that of the GA group ((OR) = 1.68, *p* <0.001). However, there was no significant difference in the average MAP and SBP between both groups in our study. Notably, hemodynamic instability during and after SA is a common intraoperative occurrence. Nonetheless, a study involving 12,929 people undergoing total hip replacements under SA showed a lower risk of cardiovascular complications and respiratory complications in spite of the occurrence of intraoperative hypotension [19]. Moreover, another meta-analysis performed by the International Consensus on Anesthesia-Related Outcomes after Surgery group (ICAROS) indicated that SA is associated with significantly lower risks of mortality, pulmonary complications, acute renal failure, deep venous thrombosis, infections, and blood transfusion than GA is among patients undergoing hip or knee arthroplasty [20]. In our clinical practice, patients with multiple co-morbidities or in a poor general condition were more likely to be administered SA because of the anesthesiologists’ preference. Consequently, the patients in our study have an older average age (SA: 64.7 years old; GA: 55 years old), and a higher percentage of them were classified as ASA III or IV in the SA group (SA: 26.5%; GA: 21.8%). Therefore, there was a potential bias in the patient groups that provoked a precipitating impact on the higher postoperative complication rate in the SA group.

We observed that patients who experienced postoperative complications were older than those who did not (68.1 ± 13.47 years vs. 56.6 ± 18.04 years, (OR) = 1.02, *p* < 0.001), which is consistent with the findings of a previous study. However, the odds ratio was only slightly higher, which does not imply an evident impact on the postoperative complications. By reviewing these previous studies, we noticed that Louise Y. Sun’s study revealed a significant increase in the duration of intraoperative hypotension with advancing age (*p* = 0.002) [8]. In addition, Dony et al. noticed, in their study of 11,304 patients with a mean age of 50.6 years, that patients who sustained mortalities within 30 days postoperatively had a higher average age of 74.5 years [11]. Older age has also been associated with a higher 30-day mortality rate following surgery [21]. Consequently, it is imperative to avoid either SBP or MAP intraoperative hypotension in order to prevent complications for elderly patients. 

### Strengths and Limitations

Our study possesses several strengths and limitations. Among the strengths, we analyzed the data extracted from an electronically recorded intraoperative blood pressure database, with readings taken at five-minute intervals for a substantial number of patients. Continuous monitoring facilitated the precise assessment of intraoperative blood pressure. Furthermore, our study ensured robust analysis with a large sample size sourced from the clinical database, thus attenuating the influence of potential confounding factors.

Nevertheless, certain limitations must be acknowledged. Firstly, our analysis did not categorize the study subjects according to the complexity of their surgical procedures, which could have potentially had varying impacts on the development of complications. Our focus was on postoperative medical complications, and we believe that the complexity of the surgical procedures may not necessarily be a significant factor warranting further consideration.

Secondly, baseline blood pressure was established using measurements taken upon admission to the ward, which might be different from the patient’s true casual blood pressure. To mitigate this potential bias, we computed the average of the blood pressure readings obtained in an emergency room or after admission. Additionally, we defined a hypotensive event for both SBP and MAP based on two consecutive blood pressure records, thus preventing any bias resulting from a single incidental hypotensive measurement.

We confirmed the diagnosis of complications by retrospectively extracting data using ICD codes in the discharge notes through cross-referencing with a clinical database, laboratory data, reports of image studies, and additional interventional procedures, representing the third limitation. The severity and treatment of the complications were not incorporated in the study design. Although most of the patients sustaining hemodynamic instability were supposed to be handled properly by anesthetists because intraoperative mortality was not reported, the lack of a pharmacological approach by the anesthetists demonstrated another limitation of this study deserving further investigation. Due to the extraction of the study material from a clinical database, we were unable to track the treatment decisions and outcomes for each individual patient. The lack of data regarding interventions for those with a higher ASA score is another limitation of this study and so we provided no management suggestions. Consequently, our discussion was restricted to examining the impact of ASA classification and hemodynamic instability on the development of medical complications.

## 5. Conclusions

In conclusion, a significant reduction in SBP during orthopedic surgery and a higher pre-operative ASA score were strongly linked to elevated complication risks. Our study highlights the importance of maintaining hemodynamic stability during operations. While hypotensive anesthesia is a common anesthetic policy used in orthopedic surgery, a cautious approach is essential to mitigate the potential complications.

## Figures and Tables

**Table 1:** Demographic characteristics of the patients.

		No Complication (n = 6090)	With Complication (n = 388)	Incidence of Complication
Age (mean ± SD)		56.6 (±18.04)	68.1 (±13.47)	
Sex	Male	2758	149	5.1%
	Female	3332	239	6.7%
Anesthesia method	GA	4719	261	5.2%
	SA	1371	127	8.5%
ASA classification	I	911	7	0.8%
	II	3967	113	2.8%
	III	1192	259	17.8%
	IV	20	9	31.3%

GA, general anesthesia; SA, spinal anesthesia; SD, standard deviation.

**Table 2:** Logistic regression analysis of perioperative parameters.

		OR	95% CI		*p* Value
			Lower	Upper	
Age		1.02	1.01	1.02	<0.001 *
Sex	Male (ref)				
	Female	1.22	0.97	1.53	0.091
Anesthesia Methods	GA (ref)				
	SA	1.68	1.34	2.09	<0.001 *
ASA	I (ref)				
	II	2.76	1.27	6.00	0.011 *
	III	17.49	7.99	38.34	<0.001 *
	IV	40.96	13.37	129.73	<0.001 *

CI, confidence interval, GA, general anesthesia; OR, odds ratio; SA, spinal anesthesia; * significant difference, *p* < 0.05.

**Table 3:** Comparison of developing hemodynamic instability and non-surgical complications between patients given general anesthesia (GA) and spinal anesthesia (SA) for operations on the lower extremities.

	GA (n = 2555)	SA (n = 1383)	*p*
non-surgical complication			0.001 *
without	2401	1262	
with	154	121	
SBP < 100 mmHg episode			<0.001 *
without	954	969	
with	1601	414	
MAP < 65 mmHg			
episode			<0.001 *
without	1612	1142	

* Significant difference, *p* < 0.05.

**Table 4:** Intraoperative hemodynamic instability and postoperative complication incidence.

Episode Counts (≥5 min)	No Complication (n = 6090)	With Complication (n = 388)	Incidence of Complications
SBP < 100 mmHg			
0	2877	191	6.2%
1	1593	86	5.1%
2	918	61	6.2%
3	407	29	6.7%
≥4	175	16	8.4%
MAP < 65 mmHg			
0	4228	260	5.8%
1	1198	73	5.7%
2	451	38	7.8%
3	117	12	9.3%
≥4	66	4	5.7%

MAP, mean arterial blood pressure; SBP, systolic blood pressure.

**Table 5:** Logistic regression analysis of intraoperative hemodynamic instability in causing non-surgical complications.

	Episode Counts (≥5 min)	OR	95% CI		*p* Value
			Lower	Upper	
SBP < 100 mmHg	0 (ref)	-	-	-	-
	1	0.82	0.60	1.13	0.224
	2	0.93	0.64	1.35	0.697
	3	1.29	0.79	2.12	0.311
	4	1.33	0.74	2.40	0.339
MAP < 65 mmHg	0 (ref)	-	-	-	-
	1	0.97	0.69	1.35	0.855
	2	1.42	0.91	2.21	0.123
	3	1.49	0.73	3.05	0.270
	4	0.92	0.38	2.57	0.869
	BP reduction ^†^				
BP variance	≤20% (ref)	-	-	-	
	>20%	1.38	1.05	1.81	0.020 *

BP, blood pressure; CI, confidence interval; MAP, mean arterial blood pressure; OR, odds ratio; SBP, systolic blood pressure; * significant difference, *p* < 0.05; ^†^ calculated as intraoperative blood pressure minus pre-operative baseline blood pressure.

**Table 6:** Comparison of operation time and blood loss among the patients without and with hemodynamic instability episodes with SBP < 100 mmHg according to whether they had operations on the upper extremities, lower extremities, or spine.

	Patients without Occurrence of SBP < 100 mmHg	Patients with Occurrence of SBP < 100 mmHg	*p* Value
Operations for upper extremities N = 993	479	514	
Mean operation time (SD) (min)	104.00 (±46.10)	111.72 (±54.69)	0.016 *
Blood loss (SD) (mL)	39.54 (±68.25)	37.55 (±81.49)	0.678
			
Operations for lower extremities N = 1816	908	908	
Mean operation time (SD) (min)	120.04 (±52.63)	143.43 (±73.75)	<0.001 *
Blood loss (SD) (mL)	128.36 (±256.50)	182.73 (±268.76)	<0.001 *
			
Operation for spine N = 170	44	126	
Mean operation time (SD) (min)	249.23 (±146.89)	354.56 (±179.02)	0.001 *
Blood loss (SD) (mL)	192.55 (±328.63)	484.82 (±696.01)	<0.001 *

SBP = systolic blood pressure; SD = standard deviation; *: *p* < 0.05, Student’s *t*-test.

**Table 7:** Comparison of operation time and blood loss among patients without and with hemodynamic instability episodes with MAP < 65 mmHg according to whether they had operations on the upper extremities, lower extremities, or spine.

	Patients without Occurrence of MAP < 65 mmHg	Patients with Occurrence of MAP < 65 mmHg	*p* Value
Operations for upper extremities	690	303	
Mean operation time (SD)	106.78 (±49.98)	110.78 (±52.76)	0.253
Blood loss (SD) (mL)	39.80 (±68.95)	35.57 (±88.31)	0.416
			
Operations for lower extremities	1298	518	
Mean operation time (SD) (min)	125.22 (±58.04)	148.06 (±77.82)	<0.001 *
Blood loss (SD)	139.24 (±253.35)	196.39 (±285.25)	<0.001 *
			
Operations for spine	105	65	
Mean operation time (SD) (min)	297.97 (±177.24)	374.66 (±167.36)	0.006 *
Blood loss (SD)	351.08 (±573.21)	503.02 (±716.82)	0.129

MAP = mean arterial blood pressure; SD = standard deviation; *: *p* < 0.05, Student’s *t*-test.

## Data Availability

The study data were extracted from the Clinical Database of Anesthesia managed by Kaohsiung Veterans General Hospital. The dataset without personal information can be provided on request in case of further clarification needed once this manuscript is published.
